# The Reelin Receptors Apoer2 and Vldlr Coordinate the Patterning of Purkinje Cell Topography in the Developing Mouse Cerebellum

**DOI:** 10.1371/journal.pone.0001653

**Published:** 2008-02-27

**Authors:** Matt Larouche, Uwe Beffert, Joachim Herz, Richard Hawkes

**Affiliations:** 1 Genes and Development Research Group and Hotchkiss Brain Institute, Department of Cell Biology and Anatomy, Faculty of Medicine, The University of Calgary, Calgary, Alberta, Canada; 2 Department of Molecular Genetics, University of Texas Southwestern Medical Center, Dallas, Texas, United States of America; Albert-Ludwigs-Universität Freiburg, Germany

## Abstract

The adult cerebellar cortex is comprised of reproducible arrays of transverse zones and parasagittal stripes of Purkinje cells. Adult stripes are created through the perinatal rostrocaudal dispersion of embryonic Purkinje cell clusters, triggered by signaling through the Reelin pathway. Reelin is secreted by neurons in the external granular layer and deep cerebellar nuclei and binds to two high affinity extracellular receptors on Purkinje cells-the Very low density lipoprotein receptor (Vldlr) and apolipoprotein E receptor 2 (Apoer2). In mice null for either *Reelin* or double null for *Vldlr* and *Apoer2*, Purkinje cell clusters fail to disperse. Here we report that animals null for either *Vldlr* or *Apoer2* individually, exhibit specific and parasagittally-restricted Purkinje cell ectopias. For example, in mice lacking Apoer2 function immunostaining reveals ectopic Purkinje cells that are largely restricted to the zebrin II-immunonegative population of the anterior vermis. In contrast, mice null for *Vldlr* have a much larger population of ectopic Purkinje cells that includes members from both the zebrin II-immunonegative and -immunopositive phenotypes. HSP25 immunoreactivity reveals that in *Vldlr* null animals a large portion of zebrin II-immunopositive ectopic cells are probably destined to become stripes in the central zone (lobules VI–VII). A small population of ectopic zebrin II-immunonegative Purkinje cells is also observed in animals heterozygous for both receptors (*Apoer2^+/−^: Vldlr^+/−^)*, but no ectopia is present in mice heterozygous for either receptor alone. These results indicate that Apoer2 and Vldlr coordinate the dispersal of distinct, but overlapping subsets of Purkinje cells in the developing cerebellum.

## Introduction

Purkinje cells in the adult cerebellum are grouped into discrete modules based on two arrays of orthogonal boundaries–transverse zones and parasagittal stripes [reviewed in 1]–[5]. Birth dating experiments demonstrate that at least a partial Purkinje cell parasagittal identity is specified at or soon after their birth in the subventricular zone of the 4^th^ ventricle (embryo age (E) 10-E13: 6–8). Heterogeneous protein expression has been observed as early as E14.5 [9]. Numerous Purkinje cell proteins are expressed in parasagittal stripe patterns in the adult cerebellum [reviewed in 1]–[3], [10], [11]. One of the best-studied examples is the adult (“late-onset”) stripe marker zebrin II/aldolase C [12], [13], which is expressed in Purkinje cells in a well-characterized pattern that is reproducible between individuals and conserved across species [14]. In addition to zebrin II, many other markers reveal parasagittal stripes of Purkinje cells in the mature cerebellum, including positive markers of the zebrin II-immunonegative subset (e.g., phospholipase Cß4 (PLCß4)–15: reviewed in 1–3). Oriented orthogonally to the parasagittal stripes are transverse expression domains [5], [16]. For example, transitions in the expression pattern of zebrin II divide the cerebellum into four interdigitated zones-the anterior zone (AZ: ∼lobules I-VIa), central zone (CZ: ∼VI–VII), posterior zone (PZ: ∼VIII-IX) and nodular zone (NZ: ∼IX/X: 5). Based on the combination of parasagittal stripes and transverse zones, the mature cerebellum can be reproducibly subdivided into several hundred discrete units [2].

Signaling by Reelin is crucial to the maturation of this complex topography. After their terminal division, Purkinje cells migrate out of the ventricular zone and accumulate in clusters in the cerebellar anlage. Reelin signaling subsequently triggers the perinatal dispersal of the Purkinje cells into the adult monolayer [e.g.], [ 17]–[19]. Reelin is a large glycoprotein secreted by Cajal-Retzius cells in the cortex and external granular layer and cerebellar nuclear neurons in the cerebellum [20], [21]. Mice null for *Reelin* show no Purkinje cell dispersal and are profoundly ataxic (*reeler* mutants: e.g., 22–24). Reelin binds to two extracellular receptors–the very low density lipoprotein receptor (Vldlr) and apolipoprotein E receptor 2 [Apoer2: 25]. Mice null for both *Apoer2* and *Vldlr* show Purkinje cell dispersal deficits nearly identical to *reeler*, indicating that both receptors are necessary for full Reelin signaling [25]. Reelin binding results in the phosphorylation of an intracellular cytosolic adaptor protein, Dab1 [26], [27] and accordingly mice null for *dab1* also have *reeler*-like Purkinje dispersal deficits [*disabled*: 19], [27], [28].

To understand better the role of Reelin signaling in the regulation of Purkinje cell migration during development, cerebella were examined from heterozygous and homozygous null animals for either *Apoer2* or *Vldlr*. Our goal in this study is to develop a map of Purkinje cell ectopia in each mutant animal by using markers that identify Purkinje cells or spatially restricted subsets of these neurons in the mature cerebellum. We have identified both unique and overlapping populations of ectopic Purkinje cells in different mutant combinations. Taken together these data suggest that each Reelin receptor directs the dispersal of distinct Purkinje cell subsets during development.

## Materials and Methods

All procedures using animals conformed to UT Southwestern IACUC approved protocols as well as *The Guide to the Care and Use of Experimental Animals* as outlined by the Canadian Council for Animal Care. *Vldlr* and *Apoer2* null mice were raised from stocks originally created through targeted deletion of each individual gene [25]. Mice were housed at room temperature (21°C) on a 12 h light/dark cycle, and genotyped by using a polymerase chain reaction assay [25].

Mice were anesthetized using isoflurane and perfused transcardially with 5–10 ml of 0.9% ice-cold saline, followed by 40 ml of freshly prepared 4% paraformaldehyde in PBS (pH = 7.2; Sigma, MO, USA). Following perfusion, the brains were removed from the skull and stored in 4% paraformaldehyde for at least 48h. For cryosectioned tissue, brains were first cryoprotected through an ascending series of sucrose solutions until they sank (10, 20, 30% sucrose w/v in PBS). The tissue was embedded by freezing in OCT (Sakura Finetek, Torrance, CA, USA) and 40 µm sections cut in either the sagittal or transverse plane. Tissue sections were stored in Millonig's solution (PBS+0.05% sodium azide) at 4°C until required.

Section immunohistochemistry was performed free floating as previously described [29]. Briefly, following peroxidase quenching and blocking in PBS containing 0.1% Triton X-100 and 10% normal donkey or goat serum (PBSTD or PBSTG, respectively), sections were incubated in primary antibodies diluted in blocking solution.

A mouse monoclonal antibody against CaBP was obtained from Swant (Bellinzona, Switzerland; Mab 300, lot #18(F); raised against chicken CaBP and specifically binds to the ^45^Ca-binding spot of calbindin D-28k (apparent molecular weight 28K, isoelectric point 4.8) in a two-dimensional gel of mouse brain homogenate (manufacturer's information)) and used here diluted 1:5000 in blocking serum. Rabbit anti-calbindin was raised against full-length recombinant CaBP (Catalog #AB1778, Chemicon, Temecula, CA, USA-used diluted 1∶5000 in PBSTG). Both anti-calbindin antibodies yielded Purkinje cell specific staining identical to that previously reported [e.g., 5], [30]. Anti-zebrin II/aldolase C is a mouse monoclonal antibody produced by immunization with a crude cerebellar homogenate from the weakly electric fish *Apteronotus* [12]: it was used directly from spent hybridoma culture medium diluted 1:5000. Rabbit polyclonal anti-heat shock protein 25 (anti-HSP25: 1∶5,000 for peroxidase immunocytochemistry; 1:1000 for immunofluorescence) was purchased from StressGen (Victoria, BC, Canada: SPA-801, lot #B111411). This antibody was raised against recombinant mouse HSP25 protein and as well, antibody specificity for HSP25 in adult murine cerebellar tissue has been previously demonstrated where antibody absorption controls using HSP25 abolished all immunostaining [see 31]. Rabbit anti-phospholipase PLCß4 (the generous gift of Dr. M. Watanabe, Hokkaido University, Sapporo, Japan: used diluted 1∶2500) was raised against a synthetic peptide representing amino acids 15–74 of mouse PLCß4 fused to glutathione-S-transferase and expressed in bacteria. Control immunohistochemistry, either with antibodies pre-absorbed with antigen polypeptides or using tissue from a PLCß4 knockout mouse, yielded no significant immunostaining [32]. An identical staining pattern was also obtained with another anti-PLCß4 antiserum, raised in guinea pig (M. Watanabe–unpublished data). Both antisera recognize single polypeptide bands of 134 kDa apparent molecular weight on Western blots of mouse [32] and human (unpublished data) cerebellar homogenates. The band is absent from Western blots of cerebellar homogenates from a PLCß4 null mouse [32], [33]. Cerebellar anti-PLCß4 immunocytochemistry has been described previously [15]. Polyclonal rabbit anti-neurogranin was raised against full-length recombinant neurogranin protein (Catalog #AB5620; Chemicon, Temecula, CA, USA-used diluted 1:5000): it recognizes Purkinje cells in the neonatal cerebellum and Golgi cells in the adult cerebellum, as previously reported [29], [34] and on Western blots of newborn or P20 cerebellar homogenates reveals a single polypeptide band of apparent molecular weight 10 kDa [29], consistent with that of neurogranin/RC3 [35]. Further details are provided in Larouche et al. (2006). Goat anti-retinoic acid orphan receptor alpha was, as indicated in the product specifications, raised against a peptide of unspecified length representing the C-terminus of the human protein (anti-ROR alpha: Catalog #C-16, Santa Cruz, Santa Cruz, CA, USA -diluted 1∶2500 in PBSTD). It recognizes a polypeptide of apparent molecular weight 67kDa on Western blots of cerebellar homogenate, consistent with ROR alpha, and does not bind to the closely related family members RORβ and γ [36]. All immunoreactivity is abolished by titration with the blocking peptide.

For peroxidase immunocytochemistry, cerebellar sections were washed 2 × 5 minutes in PBS followed by a 30 minute incubation in 0.1% H_2_O_2_ in PBS. Tissue immunoreacted with anti-PLCß4 antibodies was post-fixed in methanol (MeOH) at this stage by sequential 15 min incubations in 50% MeOH/PBS, 100% MeOH, and 50% MeOH/PBS (see Sarna et al., 2006). Next, the tissue was rinsed in PBS and blocked for 1h at room temperature in PBS containing 10% normal goat serum (Gibco, Burlington, ON, Canada) and 0.1% Triton X-100 (Sigma, St. Louis, MO, USA). Sections were incubated overnight at 4°C with gentle agitation in primary antibodies diluted in blocking solution, and washed the following morning. Following the primary incubation, sections were washed and incubated with an appropriate biotinylated secondary antibody (biotinylated IgG goat anti-mouse or IgG goat anti-rabbit; Jackson Immuno Research Labs Inc., West Grove, PA, USA-diluted 1:1,000 in blocking solution). Antibody binding sites were revealed by using the Vectastain ABC kit (Vector Laboratories Inc., Burlingame, CA., USA) with diaminobenzidine as the chromagen. Tissue sections were mounted on slides and allowed to dry for at least two hours, dehydrated through an ascending alcohol series, cleared in Histoclear (Diamed, Mississauga, ON, Canada), and coverslipped with Entellan mounting medium (BDH Chemicals, Toronto, ON, Canada).

For immunofluorescence, the tissue was blocked as above and incubated overnight with primary antibodies (mouse anti-zebrin II, rabbit anti-neurogranin, rabbit anti-HSP25, rabbit anti-PLCß4 or mouse anti-calbindin D-28k). Following incubation, the sections were washed 3×10 min in blocking solution and incubated with a fluorescently labeled secondary antibody diluted in blocking solution for 2 h at room temperature. All fluorescent secondary antibodies were obtained from Molecular Probes (Eugene, OR, USA) as Alexa fluorophore conjugates (546 nm-goat anti-rabbit IgG; 488nm-goat anti-mouse IgG). Sections were washed 3×10 minutes in PBS, mounted on slides and allowed to dry for 2 h at room temperature. The tissue was rehydrated for 5 minutes in PBS and mounted in Aqua Polymount (Polysciences, Warrington, PA, USA).

Whole mount immunocytochemistry was performed as previously described [37] except that PBS containing 5% skim milk (Nestlé Foods Inc., North York, ON, Canada) plus 0.1% Triton-X 100 (Sigma, St. Louis, MO, USA) was used to dilute the primary antibody (rabbit anti-neurogranin at 1:1000). Biotinylated goat anti-rabbit IgG (Jackson Immuno Research Labs Inc., West Grove, PA, USA) was diluted 1∶1000 in PBS containing 0.1% Triton X-100 and incubated with the cerebella overnight. Cerebella were washed with PBS (3×2 h) and incubated overnight in the ABC complex solution (Vectastain, Vector Laboratories Inc., Burlingame CA., USA). Antibody binding was revealed by using diaminobenzidine as the chromagen. Photomicrographs were captured by using a Spot CCD camera (Diagnostic Instruments, La Jolla, CA, USA). Montages were assembled in Adobe Photoshop 9.

For conventional histological examination cryosections were mounted on slides and allowed to adhere for at least two hours at room temperature. Tissue was first fixed in 10% neutral buffered formalin (BDH Chemicals, Toronto, ON, Canada) for five minutes, dehydrated in an ascending series of alcohol and xylene, and rehydrated back to distilled water. Sections were stained for 30 s in 1% w/v cresyl violet acetate in distilled water, then rinsed in several changes of distilled water until clear. Sections were re-dehydrated through an ascending alcohol series. At 100% ethanol, they were incubated 2×5 min in 20 ml ethanol containing 5 drops of 100% acetic acid, before completing the dehydration in xylene. Finally, sections were coverslipped in Entellan mounting medium (BDH Chemicals, Toronto, ON, Canada).

Lobule lengths were measured from photographs of three randomly selected vermal mid-sagittal sections from animals of each genotype (n = 3) by using Zeiss Axiovision 3.1 (Carl Zeiss, North America). The midline vermis was defined as lying between the two medial cerebellar nuclei. Boundaries between lobules were defined as the deepest part of the sulcus separating each lobule and the length of each lobule was defined as the distance between the deepest point of each sulcus. Lobule lengths were measured at the level of the Purkinje cell layer for both wild type and mutants. Lobule lengths, as well as overall cerebellar length, were compared to wild type by ANOVA and a difference of p<0.05 was considered significant.

## Results

**Figure 1:**
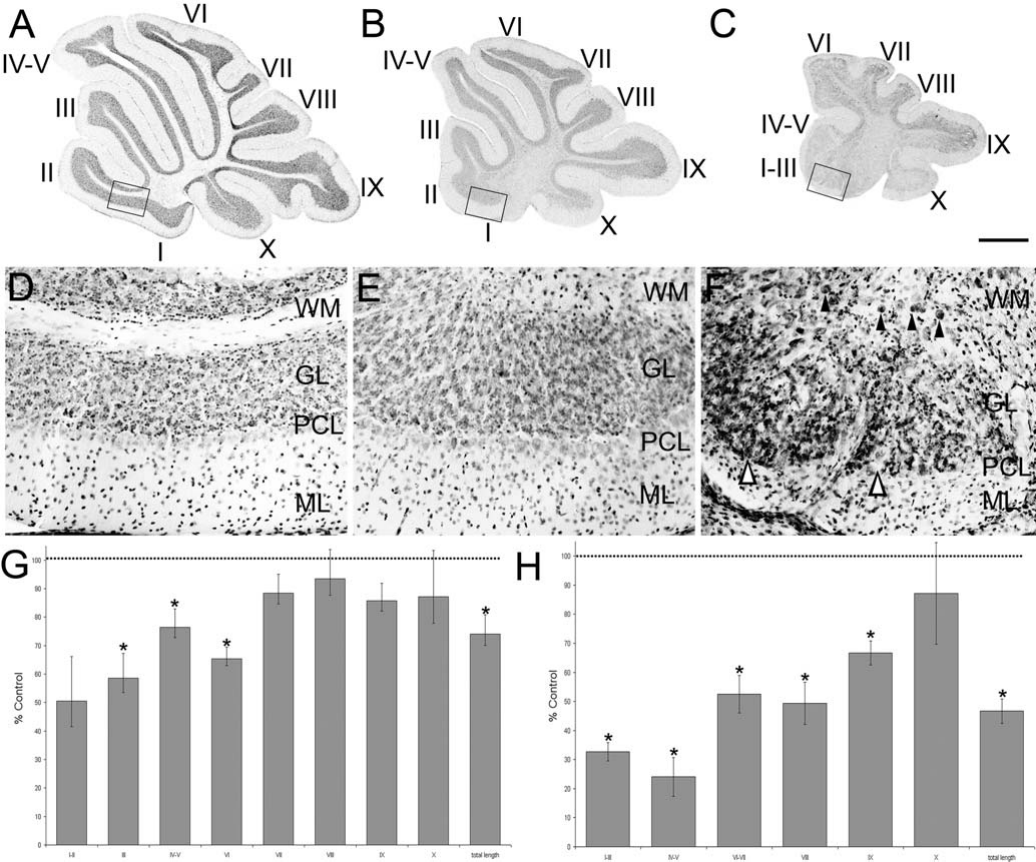
Cresyl violet staining reveals that the cerebellar cortex of *Apoer2* and *Vldlr* mutants is abnormal. Sagittal sections through the medial cerebellum of adult wild type (A, D), *Apoer2* (B, E) or *Vldlr^−^* (C, F) null animals indicate that mutant cerebella are smaller and have fewer lobules when compared to wild type mice. Higher-power views reveal that a trilaminar structure is present in both mutants and wild type (D–F) consisting of an outer molecular layer (ML), Purkinje cell layer (PCL) and inner granule cell layer (GL). White matter tracts (WM) can also be observed in each animal. High magnification views of the *Vldlr* null cerebellum reveal the presence of Purkinje cell-sized somata in the granular layer and white matter (e.g. black arrowheads–F) as well as gaps in the Purkinje cell layer (white arrowheads–F). Measurements of the length of lobules in *Apoer2* null (G) or *Vldlr* null (H) cerebella are expressed as a percentage of the length in wild-type littermates. Length measurements reveal a reduction in several areas of each mutant cerebellum. These reductions are most prominent in the anterior cerebellum of both mutants. Error bars on the graph depict SEM. Dotted line indicates the length of the equivalent lobule in wild type animals. Scale bar = 1 mm for A–C and 125 µm for D–F. * indicates p<0.05 as determined by one way ANOVA.

Previous studies have shown that the functional deletion of both *Apoer2* and *Vldlr* is necessary to block all perinatal Purkinje cell dispersal and recapitulate the *reeler* phenotype [25]. To determine if individual receptor nulls have some subtle Purkinje cell dispersal phenotypes, cerebella were examined from animals homozygous null for each individual receptor. First, sagittal sections from *Apoer2* or *Vldlr* null cerebella were stained with cresyl violet to assess lobular and cytoarchitectural abnormalities (Fig. 1). Adult wild type mouse cerebellar foliation is conventionally classified into 10 lobules (Fig. 1A) with a trilaminar structure present in all lobules-the outer molecular layer runs immediately beneath the pia and overlies the Purkinje cell monolayer and the innermost granule cell layer (Fig. 1D). *The Apoer2* null cerebellum has normal lamination and lobulation (compare Fig. 1A, D and Fig. 1B, E). However, total length measurements indicate that the null mutant cerebellum is about 25% shorter than wild type (n = 3, p<0.05; Fig. 1G). The reduced total length in the *Apoer2* null is largely attributable to smaller lobules in the rostral cerebellum: in particular lobules III–VI are 40% shorter than in control littermates (n = 3, p<0.05: Fig. 1G). In contrast, lamination of the *Vldlr* null cerebellar cortex is clearly abnormal, in that in lobules I–VII the molecular layer is approximately half as thick as in wild type (∼200 µm: Fig. 1C, F). In contrast, the molecular layer is similar to wild type thickness in the posterior lobules VIII–X (Fig. 1C, F). A closer inspection also reveals that the Purkinje cell layer is also abnormal, with numerous scattered acellular gaps (white arrowheads-Fig. 1F). Cresyl violet staining also revealed large cell somata in the white matter reminiscent of Purkinje cells (black arrowheads, Fig. 1F). However, in contrast to wild type, only 6 lobules are present in the *Vldlr* null cerebellum (Fig. 1C), and the entire *Vldlr* null vermis is only half the rostrocaudal length of the wild type (n = 3, p<0.05, Fig. 1H). Although it is not straightforward to homologize lobules in lissencephalic mutants to those in wild type, the reduction in lobule length appears to involve all lobules except lobule X. As in the *Apoer2* null cerebellum, the greatest reductions in lobule lengths are observed in the rostral vermis, which is 60–70% shorter than in littermate controls (Fig. 1H). However, in contrast to the *Apoer2* null cerebella, the posterior lobules are also reduced in length: putative lobule VI/VII, which is fused in *Vldlr* nulls, is only 50% of the length of the combined lobules in wild type, and lobules VIII and IX are also reduced 40–50% in total length (Fig. 1H).

### Purkinje cell ectopia is predominantly restricted to zebrin II-immunonegative Purkinje cells in the adult *Apoer2* homozygous null cerebellum

**Figure 2:**
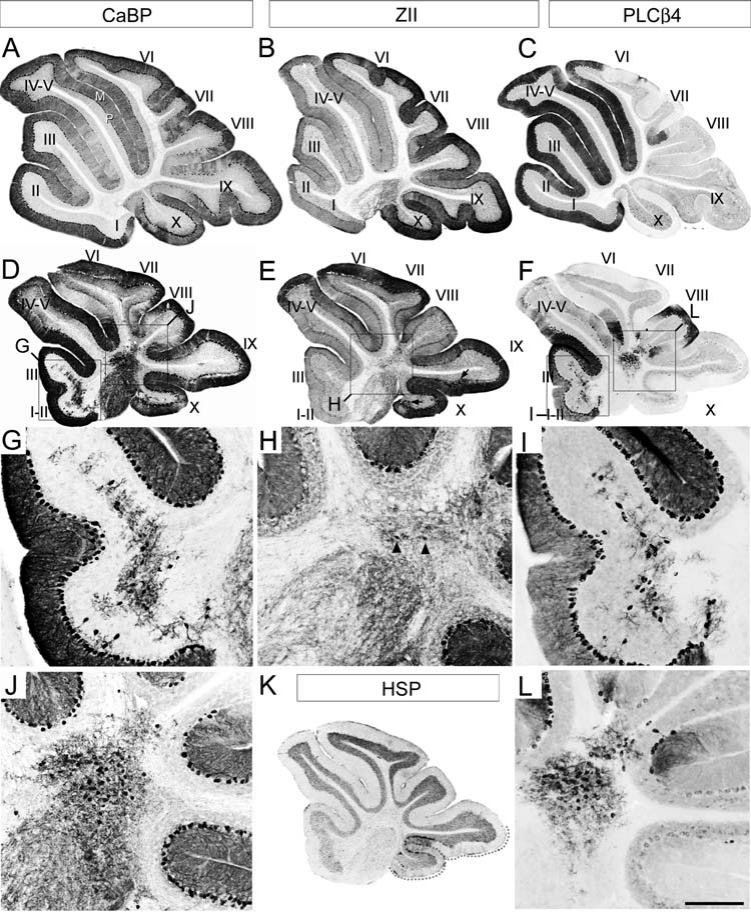
Adult *Apoer2* null cerebella have Purkinje cell ectopia that is largely restricted to zebrin II-immunonegative cells. Sagittal sections are taken from either adult wild type (A–C) or *Apoer2* null (D–L) cerebella. Cerebella have been immunostained with antibodies against calbindin (CaBP-A, D, G, J), zebrin II (ZII-B, E, H), phospholipase Cß4 (PLCß4-C, F, I, L) or heat shock protein 25 (HSP25-K) to reveal immunopositive Purkinje cell bodies in the Purkinje cell layer (P) as well as their dendrites located within the molecular layer (M). Sections from the *Apoer2* null cerebellum are serial sections (zebrin II-calbindin-PLCß4) while wild type sections are not. Boxes in D–F indicate areas where higher-magnification pictures are presented below. High-magnification panels (G, H, I, J, L) illustrate the presence of discrete groups of ectopic Purkinje cells in the white matter of the *Apoer2* null cerebellum, as identified with CaBP-immunostaining (G, J).The absence of zebrin II immunoreactivity in these cells (H) indicates that the predominant phenotype of Purkinje cells in the white matter of these mutants is ZII-/PLC ß4+ (I, L). Black arrows in E point to areas in lobules IX and X where Purkinje cells are misaligned within the Purkinje cell monolayer. Arrowheads in H point to the rare occurrence of zebrin II immunopositive Purkinje cells in the ectopic clusters. K–HSP25 immunoreactivity is revealed in Purkinje cells throughout the NZ (dotted line). Roman numerals indicate lobules. Scale bar in L = 1 mm for A–F and 250 µm for E–L.

To understand why the adult *Apoer2* null cerebellum is reduced in rostrocaudal length, sagittal sections were immunostained by using several Purkinje cell markers (Fig. 2). First, the pan-Purkinje cell marker calbindin [e.g. 30] was used to locate all Purkinje cells in the *Apoer2 null* cerebellum. Calbindin immunoreactivity is observed throughout all Purkinje cells, including their dendrites in the molecular layer (e.g. M–Fig 2A), the somata in the Purkinje cell layer (e.g. P–Fig. 2A), and the axons in the white matter tracts, in a fashion identical to wild type (Fig. 2A, D). Calbindin immunoreactivity also reveals two distinct populations of ectopic Purkinje cells located in the white matter of the *Apoer2* null cerebellum (Fig. 2G, J)-one sparsely distributed through the white matter of lobules I–III (Fig. 2G) and a second, forming a densely packed cluster located dorsally within the white matter of the cerebellar core (Fig. 2J: see also 36).

To characterize the phenotype(s) of the ectopic Purkinje cells in the *Apoer2* null cerebellum, sagittal sections serial to those immunostained with calbindin above were immunoperoxidase stained using antibodies against several markers capable of revealing the parasagittal organization of Purkinje cells. These markers included zebrin II (Fig. 2B, E, H), phospholipase C ß4 (PLCß4-Fig. 2C, F, I, L) and heat shock protein 25 (HSP25-Fig. 2K). Zebrin II immunostaining in the adult wild type cerebellum reveals a reproducible pattern of parasagittal stripes ([e.g.], [37, 39]; Fig. 2B). Importantly, normal Purkinje cell positioning is not required for restricted zebrin II expression as it is expressed in parasagittal subsets of ectopic Purkinje cells in several dispersal mutants (e.g., *disabled*-19; *weaver*–40; *cerebellar deficient folia*-41). In general, anti-zebrin II immunostaining of the *Apoer2 null* cerebellum appears normal, suggesting that few zebrin II-immunopositive Purkinje cells are ectopic (Fig. 2B, E). Occasionally, ectopic zebrin II-immunopositive Purkinje cells were observed in the cluster located ventral to lobule VI–VII and identified with calbindin immunostaining (e.g., arrowheads-Fig. 2 H). The largest concentration of ectopic zebrin II-positive Purkinje cells is in the nodular zone (NZ = ventral lobule IX and X–Fig. 2K dotted line; 5) of the *Apoer2* null (e.g. arrows, Fig. 2E). These ectopic zebrin-expressing Purkinje cells are confined to the NZ since there are few ectopic Purkinje cells observed in the neighbouring posterior zone ( = PZ-lobules VIII and dorsal IX). Reproducible clusters of Purkinje cells are misaligned within the Purkinje cell layer in the NZ (Fig. 2E–arrows; 3Q–R).

Finally we conducted HSP25 immunolabeling using sagittal sections from the *Apoer2* null cerebellum in order to better understand the organization of transverse zones in these mutants. HSP25 immunoreactivity in the vermis marks a subpopulation of zebrin II-immunopositive Purkinje cells in both the NZ and CZ [31]. The NZ of the *Apoer2* null cerebellum resembles wild type and therefore appears unaffected (Fig. 2K–dotted line). HSP25 in the *Apoer2* null is expressed in Purkinje cell stripes extending throughout lobules VI–VII in a parasagittal pattern similar to wild type (data not shown). However, while the rostral limit of HSP25 expression in the wild type cerebellum normally ends in the anterior face of lobule VI within the primary fissure [31], the furthest anterior that we have detected Purkinje cell HSP25 immunoreactivity in the *Apoer2* null CZ is the caudal face of lobule Via (data not shown). This caudal displacement of the limit of HSP25 expression in the *Apoer2^−/−^* CZ suggests that cerebellar lobulation has shifted with respect to the rostral boundary of the CZ.

The calbindin/zebrin II expression data suggest that most ectopic Purkinje cells in the white matter of the *Apoer2* null cerebellum are zebrin II-immunonegative. To confirm this hypothesis, sections serial to those immunostained with calbindin above were immunostained by using antibodies against PLCß4, a positive antigenic marker of the zebrin II-immunonegative Purkinje cells (15; Fig. 2F, I, L). PLCß4 immunoreactivity reveals numerous ectopic Purkinje cells both in the AZ and CZ, located in clusters with the same mediolateral locations as those identified by using calbindin immunostaining (compare Fig. 2G and Fig. 2I; Fig. 2J and Fig. 2L). As expected, since the NZ is almost completely zebrin II-immunopositive in the wild type, no PLCß4 immunolabeling is present in lobules IX/X of the *Apoer2* null (Fig. 2F).

**Figure 3:**
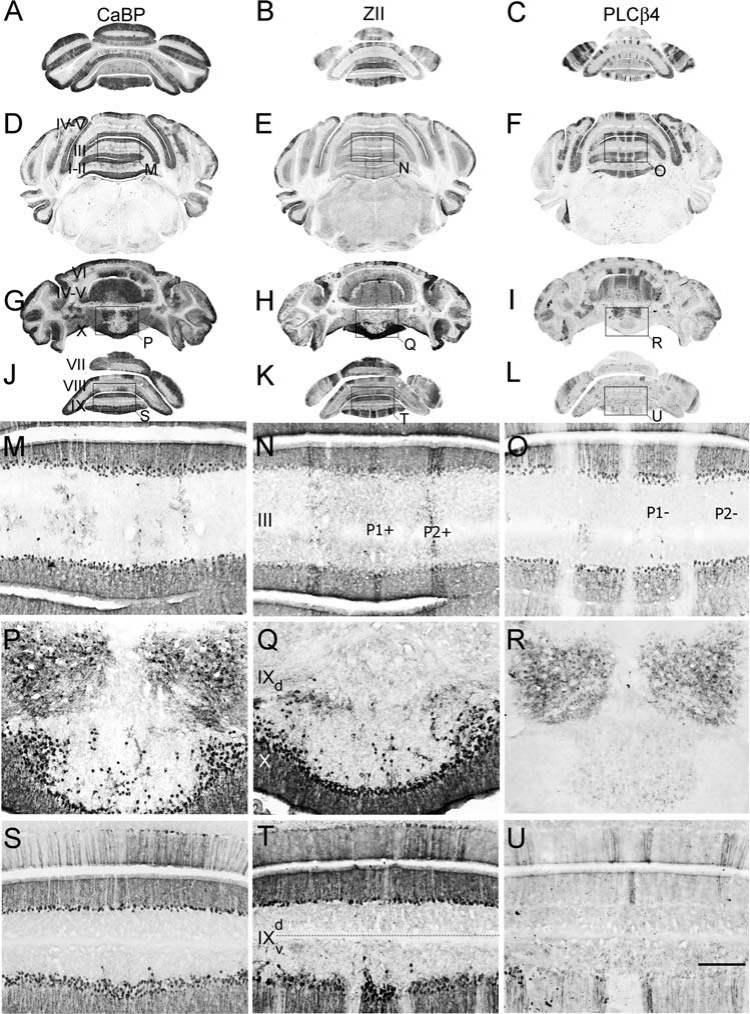
Purkinje cell ectopia is aligned with parasagittal organization in the *aporER2* null cerebellum. A series of transverse cryosections through adult wild type (A–C) or *Apoer2* null (D–R) cerebella immunostained with antibodies against calbindin (CaBP-A, D, G, J, M, P), zebrin II (ZII-B, E, H, K, N, Q) or phospholipase Cß4 (PLCß4-C, F, I, L, O, R). Sections from the *Apoer2* null cerebellum are serial (zebrin II-calbindin-PLCß4). Boxes in D–I indicate areas where higher-magnification photomicrographs are presented below as indicated by the label. High power views of anterior cerebella immunostained with CaBP (M) reveal that ectopic Purkinje cells are restricted to discrete parasagittal domains. Immunostaining with ZII and PLCß4 in neighboring sections reveals that these ectopic cells are composed of discrete and non-overlapping stripes of ectopic Purkinje cells (N, O). Ectopic Purkinje cells in the PZ (lobules VIII and dorsal IX) are from the zebrin II-negative subset (compare Q, R). Purkinje cell ectopia in the nodular zone (NZ) is limited to ZII positive Purkinje cells (S–U). Dotted line in panel T marks the approximate dorsoventral boundary within lobule IX between the posterior zone (including the dorsal half of lobule IX) and nodular zone (ventral IX and X). The transition between PZ and NZ in lobule IX highlights the differing characteristics of ectopic Purkinje cells in the NZ (misaligned ZII-positive) and PZ (ectopic clusters of ZII-negative Purkinje cells). P1+ and P2+ denote zebrin II-positive/PLCß4-negative Purkinje cell stripes, while P1- and P2- label zebrin II-negative/PLCß4-positive Purkinje cell stripes. Roman numerals indicate lobules. Scale bar in R = 1 mm for A–I and 250 µm for J–R.

Next, serial transverse cryosections taken from adult *Apoer2* null animals were immunostained to try to assign the ectopias to specific Purkinje cell stripes (Fig. 3). As in sagittal sections, transverse cryosections through wild type cerebella reveal prominent calbindin immunoreactivity in the molecular and Purkinje cell layers (Fig. 3A). Likewise, in transverse sections from *Apoer2* null animals immunostained with calbindin, reaction product was deposited both in Purkinje cells of the Purkinje cell layer (Fig. 3D, G, J) and in ectopic cells in the white matter (Fig. 3M, P, S). Three distinct populations of ectopic Purkinje cells could be identified. The first is concentrated in the anterior lobe (mainly lobules I–III; e.g., Fig. 3M). However, rather than the random, scattered distribution suggested by the sagittal sections (e.g., Fig. 2D), the ectopic Purkinje cells align rostrocaudally into three bilateral pairs of stripes, each 2–4 cells wide and separated from one another by ∼300 µm (Fig. 3M). A second population of ectopic Purkinje cells forms bilateral clusters approximately 450 µm in diameter located in the white matter dorsal to lobule X, each beginning 75 µm from the cerebellar midline (Fig. 3 J, S). A third population of ectopic Purkinje cells is observed in the granular layer and white matter of the NZ, as was seen in the sagittal sections (Fig. 3G, P).

Zebrin II immunostaining of wild type transverse sections reveals a symmetrical, highly reproducible pattern of parasagittal Purkinje cell stripes (zebrin II-immunopositive stripes are numbered P1+ to P7+; zebrin II-immunonegative stripes are numbered P1- to P6-: see 12, 37, 39; e.g., Fig. 3B). Similarly, in the *Apoer2* null zebrin II immunostaining reveals a pattern of immunoreactive Purkinje cells in transverse sections that is reminiscent of wild type (Fig. 3, E, H, K). Although some ectopic zebrin II-positive Purkinje cells are observed in the anterior zone of the *Apoer2* null cerebellum (Fig. 3N), the majority of ectopic zebrin II-immunopositive cells are located in the NZ, with no overt restriction to parasagittal stripes (Fig. 3Q). As indicated in sagittal sections (Fig. 2E) the ectopic zebrin II-positive Purkinje cells are only in the NZ (e.g. Fig 3T) and absent from the PZ. The ectopic zebrin II-immunopositive cells that are present in the AZ were located beneath the P2+ stripes (Fig. 3N) and are likely misaligned Purkinje cells that failed to complete their normal dispersal.

As in the sagittal sections, PLCß4-immunocytochemistry identified two groups of ectopic Purkinje cells–one in the white matter of the AZ lobules I–III (Fig. 3F, O) and a second a bilateral pair of Purkinje cell clusters dorsal to lobule X (Fig. 3I, L, R, U). Based on the position and phenotype of ectopic AZ Purkinje cells (i.e., zebrin II-/PLCß4+), they are likely cells that failed to disperse fully into the P1- stripes (Fig. 3O). The destination of the second group of ectopic Purkinje cells is less clear, as the normal NZ consists completely of zebrin II-immunopositive/PLCß4-immunonegative Purkinje cells (Fig. 3R). However given their proximity to lobule IX and the fact that dorsal aspect of this lobule constitutes a portion of the posterior zone (PZ), which is enriched in zebrin-negative Purkinje cells, suggests that these cells may have been destined to populate the PZ.

### A large population of ectopic Purkinje cells is observed in adult *Vldlr* null cerebella, accompanied by abnormal lobulation

**Figure 4:**
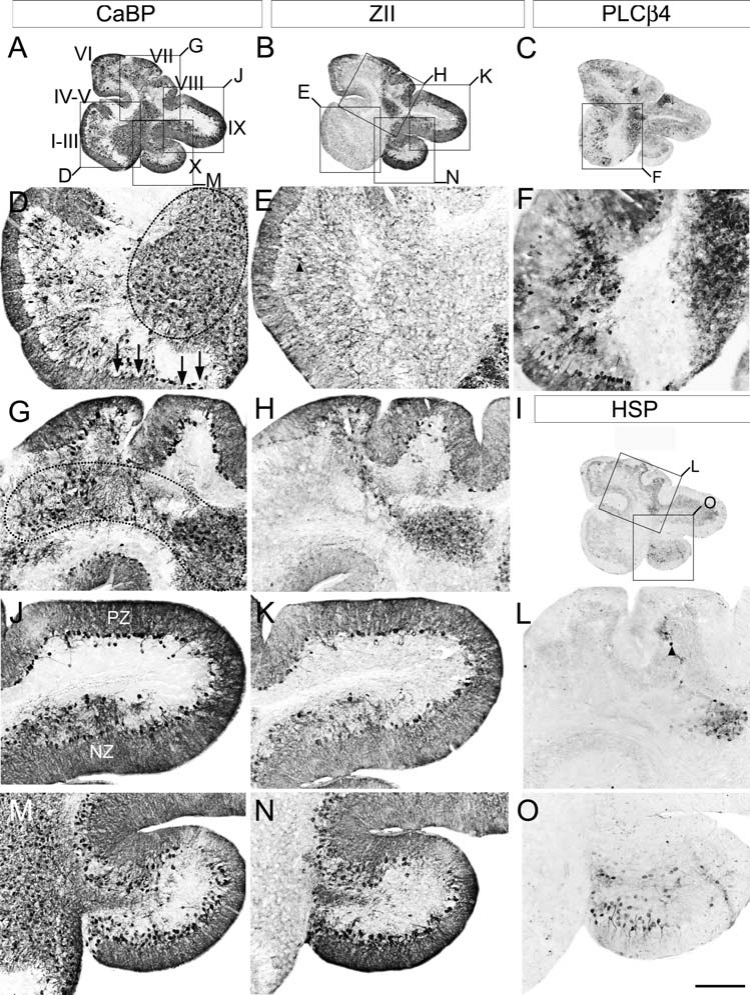
Immunostaining of sagittal sections from adult *Vldlr* null cerebella reveals that Purkinje cell ectopia includes cells from both zebrin II-immunonegative and -immunopositive subsets. A series of sagittal cryosections is illustrated from the vermis of adult *Vldlr* null cerebella immunostained for calbindin to reveal the location of all Purkinje cells (CaBP-A, D, G, J, M), as well as zebrin II (ZII-B, E, H, K, N), phospholipase C ß4 (PLCß4-C, F), or heat shock protein 25 (HSP25-I, L, O) to reveal the location of select subsets of Purkinje neurons. All four markers reveal that some Purkinje cells are correctly located within the Purkinje cell monolayer at the cerebellar cortex (e.g. between arrows–4D) as well as ectopically within the cerebellar white matter (e.g. 4D, G–dotted circles). The transition from posterior zone ( = PZ-lobule VIII and dorsal IX-Fig. 4J) into nodular zone ( = NZ-ventral IX and X–Fig. 4J) is revealed in the form of Purkinje cell ectopia (J, K). In the dorsal aspect of lobule IX Purkinje cells are restricted to a monolayer, with some ectopic cells located in the lobule white matter (J, K). In the ventral aspect of IX, the area of transition between the PZ->NZ, is highlighted by Purkinje cells misalignment and this misalignment extends the length of the NZ to include lobule X (Fig. 4M, N). Roman numerals denote putative lobule assignments. Scale bar = 1 mm for A–C and 250 µm for D–O.

**Figure 5:**
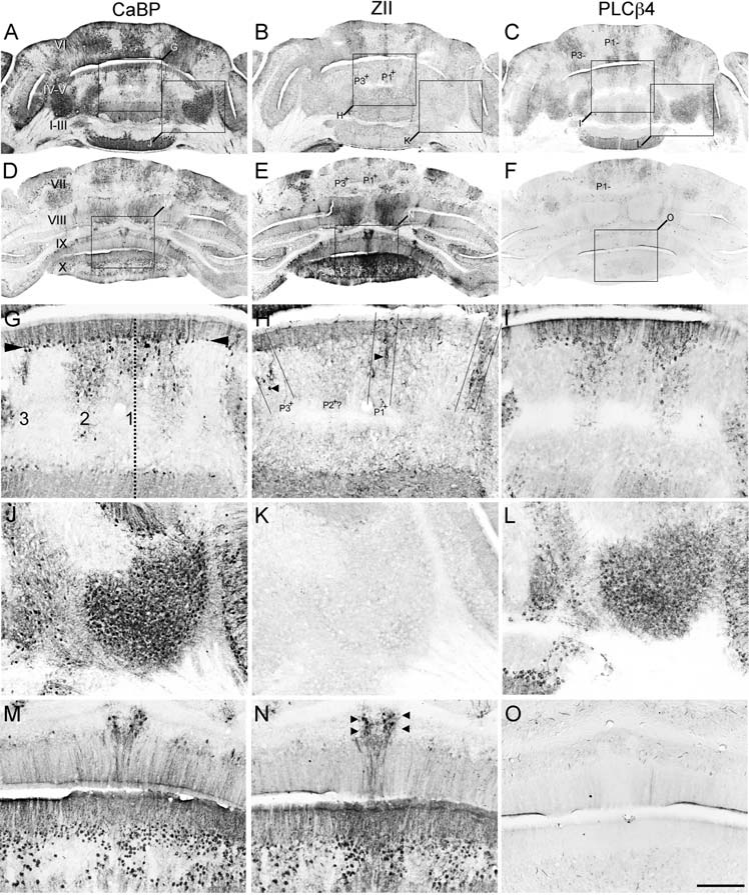
Purkinje cell ectopia in the *Vldlr* null cerebellum is parasagittally organized. Serial transverse cryosections through adult *Vldlr* null cerebellum immunostained with calbindin to reveal the location of all Purkinje cells (CaBP-A, D, G, J, M), or with zebrin II (ZII-B, E, H, K, N) phospholipase C ß4 (PLCß4-C, F, I, L, O) antibodies to reveal the location of parasagittal subsets of Purkinje cells. Boxes in A–F mark areas of higher magnification presented in the photomicrographs beneath as indicated by the letter on the corner of the box. Cells immunopositive for any of these three markers (CaBP, ZII, or PLCß4) are observed properly positioned within the Purkinje cell monolayer at the cerebellar cortex however numerous ectopic cells are also distributed throughout the cerebellar intralobular white matter. Most ectopic Purkinje cells in the anterior cerebellum are ZII-/PLCß4+ (5G–I). Some ZII-expressing Purkinje cells were observed in the granular layer (i.e. arrowheads–4E, 5H) and these ectopic Purkinje cells align in rough parasagittal stripes consistent with the overlying Purkinje cell topography in the cerebellar cortex (dotted lines-5H). M–O: high power views of ventral lobule IX (NZ) reveals that misaligned Purkinje cells are arranged into parasagittally-restricted groups, that are all zebrin II-positive (N). P1+, and P3+ mark zebrin II-immunopositive/PLCß4-immunonegative stripes and P1- and P2- mark zebrin II- immunonegative/PLCß4-immunopositive stripes. Arrowheads in G mark the Purkinje cell layer, the vertical dotted line denotes the midline, and numbers denote the location of ectopic clusters of Purkinje cells within the lobular white matter. Roman numerals denote cerebellar lobules. Scale bar in O = 1 mm for A–F and 250 µm for G–O.

Calbindin immunostaining of sagittal sections from adult *Vldlr* null cerebella revealed similar robust immunoreactivity in Purkinje cells throughout the rostrocaudal extent of the *Vldlr* null cerebellum (Fig. 4A). Consistent with our observations using cresyl violet staining (Fig. 1), multiple gaps were also observed in the Purkinje cell monolayer (arrows, Fig. 4D). The Purkinje cell layer is best aligned in putative lobules VIII and dorsal IX ( = PZ) where it is often appropriately a single cell layer thick, but even here misalignment is common, particularly near the boundaries with neighboring zones (Fig. 4J). Elsewhere, large numbers of calbindin-immunopositive, improperly dispersed Purkinje cells were observed, both scattered in the intralobular white matter (e.g. 4G) and in large clusters near the cerebellar nuclei (Fig. 4D). In particular, a large densely packed mass of Purkinje cells lies approximately midway between the anterior lobe and lobule X (dotted oval, Fig. 4D), and a second, loosely-packed cluster is found in the dorsal cerebellar white matter adjacent to the putative PZ (dotted oval, Fig. 4G). Calbindin-immunostained sagittal sections also reveal additional clusters of ectopic Purkinje cells in the paravermis and hemispheres (data not shown; see Fig. 5).

Sagittal sections serial to the calbindin immunostained sections above, immunoreacted with anti-zebrin II, suggest that few zebrin II-immunopositive Purkinje cells are ectopic in the anterior lobe of the *Vldlr* null cerebellum (Fig. 4E), but many were observed in an ectopic cluster immediately ventral to lobules VIII–IX (Fig. 4H), and in ventral lobule IX and lobule X (i.e., the NZ-Fig. 4K, N). Finally, *Vldlr* null sagittal sections serial to the calbindin-immunostained sections above and immunostained for PLCß4 (Fig. 4C, F) also reveal immunopositive Purkinje cells in a cluster in the central cerebellum immediately ventral to lobule VIII (Fig. 4F) and scattered throughout the white matter of the anterior lobe (Fig. 4F). A zebrin II-immunopositive ectopic cluster is located immediately dorsal (e.g., Fig. 4H). Given the proximity of the zebrin II-immunopositive cluster to the overlying dorsal cerebellum–an area that is predominantly zebrin II-immunopositive in the wild type (i.e., = CZ: 5, 39)-the data suggest that these zebrin II-immunopositive cells were destined to constitute a portion of the CZ.

As described above, the CZ and NZ are delineated by the limits of expression of the Purkinje cell stripe marker HSP25 [31]. HSP25 immunoreactivity in the *Vldlr* null cerebella is confined to two groups of Purkinje cells-one located in the dorsal cerebellum, and a second group restricted to the ventral face of lobule X (Fig. 4I, L, O). The Purkinje cell layer in the dorsal cerebellum of the *Vldlr* null mouse (the putative CZ) contains few HSP25-immunopositive Purkinje cells (e.g., arrowhead-Fig. 4L). The bulk of the HSP-immunopositive Purkinje cells are located in an ectopic cluster beneath the cerebellar cortex (Fig. 4I, L). This observation concurs with those made from zebrin II immunostained tissue and is consistent with the hypothesis that Purkinje cells from the CZ are ectopic in the *Vldlr* null cerebellum. The second group of HSP25-immunopositive Purkinje cells is located almost entirely within putative lobule X. This population likely contributes to the stripes of HSP25-imunopositive Purkinje cells normally observed in the NZ (Fig. 4O).

The parasagittal patterning of the *Vldlr* null cerebellum was examined to identify the putative stripe destinations of the ectopic Purkinje cells. Anti-calbindin immunolabeling of transverse cryosections of adult *Vldlr* null cerebella (Fig. 5A, D, G, J, M) reveals that most Purkinje cells are correctly located in a monolayer (Fig. 5G-arrowheads). However, calbindin immunoreactivity also reveals several reproducible, bilateral clusters of ectopic Purkinje cells (Fig. 5G, J, M). The ectopic Purkinje cells in the white matter of lobules I-V (AZ) align into three parasagittal stripes-one pair 50 µm either side of the midline, a second pair 200 µm lateral of the midline, and a third pair in the lateral vermis, 600 µm on either side of the midline (1, 2, 3-Fig. 5G). In addition, a large ectopic Purkinje cell cluster (∼600 µm in diameter) lies in the paravermis of the AZ (Fig. 5J). In the CZ (lobules VI–VII) of the *Vldlr* null cerebellum, calbindin-immunostaining reveals three symmetrically distributed cluster-pairs of ectopic Purkinje cells in the granular layer and white matter (Fig. 5D). More caudally, calbindin immunostaining in the PZ appears normal (Fig. 5D). In the NZ, calbindin-immunoreactivity reveals frequent ectopic Purkinje cells in the white matter of lobule X as well as several small, reproducible stripes of Purkinje cells misaligned within the Purkinje cell monolayer (Fig. 5N-arrowheads).

Anti-zebrin II immunostaining of the anterior vermis of the *Vldlr* null revealed three strongly immunoreactive stripes symmetrically distributed about the cerebellar midline (Fig. 5B). However, the pattern is clearly abnormal. As in wild type [e.g., 37] the ∼50 µm wide zebrin II-immunopositive P1+ stripe straddles the midline but in contrast, the ∼50 µm wide P2+ stripe normally located approximately 450 µm either side of the midline is absent (Fig. 5H). Rather, a prominent pair of immunopositive stripes is located ∼100 µm more lateral, approximately consistent with the location of the normal P3+ zebrin II-immunopositive stripe, suggesting that P2+ may be entirely absent (P2+?-Fig. 5H). Each stripe is a mixture of correctly positioned Purkinje cells as well as several cells located ectopically in the lobular white matter but aligned beneath the normally positioned P1+ and P3+ stripes (e.g., Fig. 5H-arrowheads). As in the wild type vermis, P1+ and putative P3+ extend the full length of the AZ in the *Vldlr* null (Fig. 5B, H). However, both stripes apparently project much further caudally in the *Vldlr* null cerebellum than in wild type: in wild type P1+ to P3+ do not extend caudally beyond lobule VIa [37], [39] whereas in the *Vldlr* null they extend halfway through the dorsal aspect of the cerebellum, ending caudally in putative lobule VIII (Fig. 5B, E).

More caudally, Purkinje cells in the *Vldlr* null PZ (lobules VI–VII) express zebrin II in a pattern that is reminiscent of wild type, with broad zebrin II-immunopositive stripes separated by narrow stripes of zebrin II-immunonegative Purkinje cells (Fig. 5E). Finally, as in wild type, zebrin II is expressed in all Purkinje cells of the *Vldlr* null NZ, where they form a mixture of cells aligned normally and those located ectopically (data not shown, Fig. 5N). The ectopic Purkinje cells in the NZ take two forms–scattered randomly throughout the granular layer and intralobular white matter, and in reproducible clusters misaligned from the Purkinje cell layer (arrowheads-Fig. 5N). Co-immunolabeling of transverse sections from the *Vldlr* null cerebellum using calbindin and HSP25 antibodies confirmed that most HSP25-expressing Purkinje cells are misaligned. However, many non-HSP25-immunoreactive Purkinje cells are also misaligned in the NZ indicating that this phenotype is not restricted to the HSP25-expressing Purkinje cells alone (data not shown).

**Figure 6:**
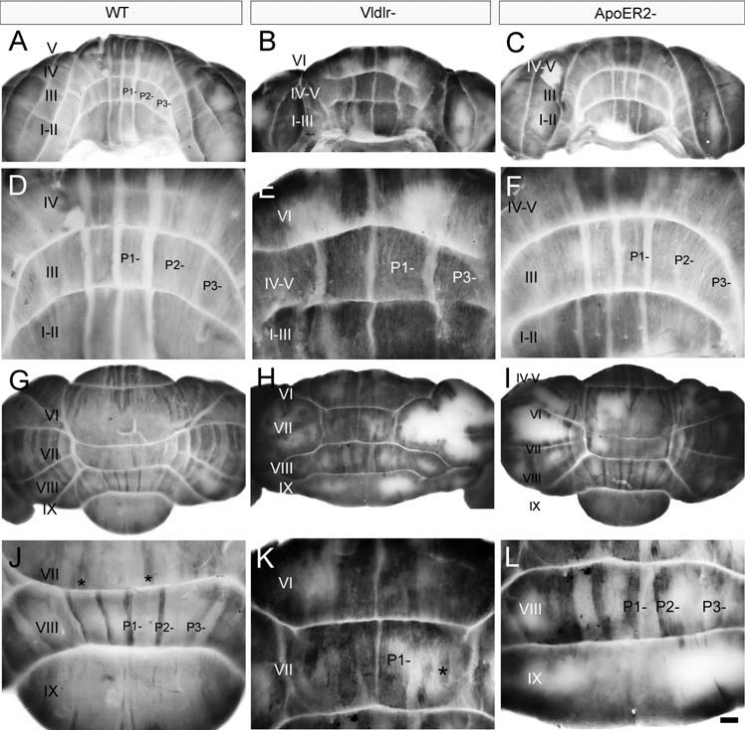
PLCß4 immunostaining in *Vldlr* null whole mounts reveal patterning changes. Whole cerebella from wild type (WT-A, D, G, J), *Vldlr* null (Vldlr^−^-B, E, H, K) or *Apoer2* null (ApoER2^−^-C, F, I, L) cerebella immunostained with anti-PLCß4 antibodies. P1+, and P2+ mark zebrin II-immunopositive/PLCß4-immunonegative stripes while P1-, and P2- mark immunonegative/PLCß4-immunopositive stripes. The stripes are subtly altered in more dorsal lobules of the Vldlr- cerebellum. Specifically, as the P1- stripes enter putative lobule VI they narrow to ∼400 µm, while the P3- stripe remains roughly the same width as the AZ (∼600 µm) but veers sharply towards the hemispheres (E). PLCß4 whole mount immunostaining of the *Apoer2* null cerebellum (C, F, I, L) reveals a parasagittal stripe pattern that is largely reminiscent of wild type and relatively unchanged despite the Purkinje cell ectopia observed inside the cerebellum (Fig 2, 3). Roman numerals (I–X) indicate lobules. Scale bar in L = 200 µm and applies to A–C, and G–I;  = 500 µm for G–F, and J–L.

Finally, *Vldlr* null transverse cryosections and cerebellar whole mounts were immunolabeled with anti-PLCß4 antibodies (Fig. 5C, F, I, L, O, and Fig. 6). PLCß4 immunostaining in the wild type AZ reveals three pairs of thick stripes of Purkinje cells (P1-: 400 µm wide; P2- and P3- each 1200 µm wide: Fig. 6A, D). Interestingly, in lobules I–V of the *Vldlr* null cerebellum, PLCß4 immunoreactivity only reveals two pairs of immunopositive Purkinje cell stripes–the putative P1- and P3- (Fig. 5I; Fig. 6B, E). Moreover, the medial stripe pair of PLCß4-expressing Purkinje cells are each approximately 600 µm wide (i.e., ∼50% wider than in wild type littermates), whereas each member of the lateral stripe pair, situated in the *Vldlr* null paravermis, is ∼800 µm wide (i.e., ∼50% narrower: Fig. 5I; Fig. 6B, E). As in the wild type, both the P1- and P3- stripes extend the length of the anterior vermis (Fig. 6B, E). In addition, several discrete ectopic clusters of immunoreactive Purkinje cells are found in the white matter (Fig. 5I, L). For example, in the anterior cerebellum, reproducible clusters align with the medial edges of P1- and P3- which extend into putative lobules VI–VII (Fig. 5C), and a third ectopic cluster, approximately spherical and 600 µm in diameter, is located in the paravermis, centered ∼1.5 mm from the midline (Fig. 5L).

In the posterior vermis of the wild type cerebellum, PLCß4-immunopositive stripes do not extend caudally beyond the rostral aspect of lobule VI (15; Fig. 6G). In the *Vldlr* null cerebellum both P1- and P3- extend caudally into putative lobules VI and VII (Fig. 6H). As in lobules I–V, PLCß4-immunopositive Purkinje cells underlying P1- and P3- in lobules VI and VII are also observed in the *Vldlr* null cerebellum (Fig. 5F, I: data not shown for P3-). The caudal aspect of lobule VII in the wild type cerebellum houses a pair of 150 µm wide stripes that appear to be anterior extensions of the P2- stripe from the PZ (denoted by *, Fig. 6J). The *Vldlr* null cerebellum also displays a pair of ∼100 µm wide PLCß4 stripes project rostrally from lobule VIII into lobule VII, as in wild type (denoted by *, Fig. 6K). Finally, three pairs of 50–100 µm wide stripes extend the length of the PZ in both *Vldlr* null and wild type cerebella. Although the stripes are about twice wide as their putative homologs in the *Vldlr* null cerebellum, the immunonegative territories between them are approximately the same widths in both (compare Fig. 6J, L). Aside from a few immunopositive Purkinje cells in the dorsal aspect of lobule IX-the caudal limit of the PZ, PLCß4 immunoreactivity in Purkinje cells is absent from the NZ of both wild type and *Vldlr* null cerebella (Fig. 5F, O; Fig. 6J, L).

We also conducted PLCß4 immunohistochemistry on whole adult *Apoer2* null cerebella (Fig. 6C, F, I) and found that despite the extensive Purkinje cell ectopia observed in immunostained sections (Fig. 2 and 3), the pattern of PLCß4 expression in the whole *Apoer2* null vermis is very similar to that in wild type. For example, prominent immunoreactive stripes of Purkinje cells are present in the Apoer2-/- AZ and PZ, while no immunoreactivity was observed in the CZ and NZ (Fig. 6C, F).

### Ectopic Purkinje cells segregate outside of the deep cerebellar nuclei

**Figure 7:**
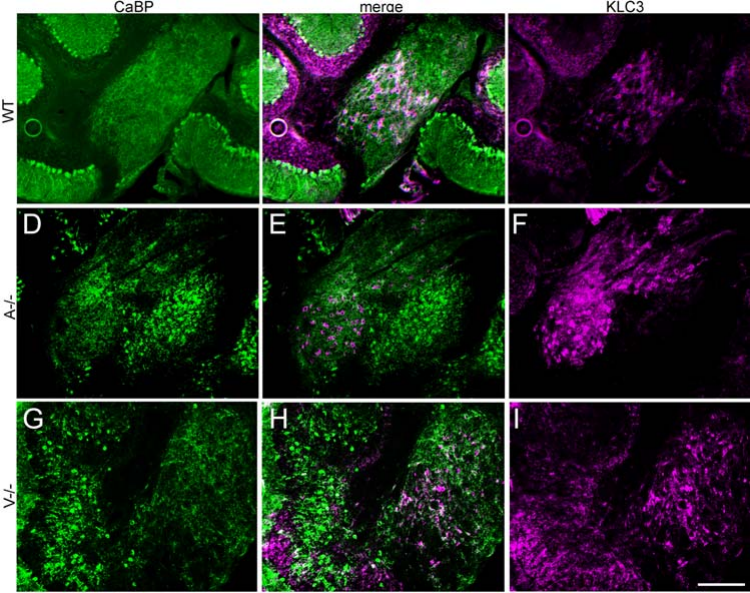
KLC3 immunolabeling reveals that ectopic Purkinje cells do not intermingle with the deep cerebellar nuclei. Sagittal cryosections from adult cerebella immunofluorescence labeled by using antibodies against KLC3 (magenta) to identify cerebellar nuclear neurons and calbindin (green) to identify Purkinje cells in wild type (A–C), *Vldlr* (D–F), or *Apoer2* (G–I) null cerebella. Merged images reveal that ectopic Purkinje clusters lie near to, but outside of the deep cerebellar nuclei in both *Apoer2* (A-/-) and *Vldlr* (V-/-) null mice. KLC-immunopositive cerebellar nuclear neurons are surrounded by the calbindin-immunoreactive axons of the Purkinje cells and these observations are consistent with previous studies of deep cerebellar nuclear neuron labeling (Chung et al., 2006). Wild type littermates (WT) have few ectopic Purkinje cells. Scale bar = 250 µm.

Purkinje cells and cerebellar nuclear neurons arise during development from the neuroepithelium of the fourth ventricle and rhombic lip, respectively [6], [7], [20], from which they migrate and accumulate in clusters [e.g., 42]. Since the ectopic Purkinje cell clusters in *Apoer2* and *Vldlr* null animals lie near to the cerebellar nuclei it is important to differentiate the between the two, and identify possible intermingling. To this end, double immunofluorescent labeling was conducted using anti-calbindin (Purkinje cell-specific) and anti-KLC3, a cerebellar marker specific for cerebellar nuclear neurons [43]. Double-immunolabeled sections reveal numerous ectopic Purkinje cells near the cerebellar nuclei (Fig. 7) but in both *Apoer2* and *Vldlr* null cerebella, Purkinje cells and cerebellar nuclear neurons form distinct, non-overlapping clusters.

### Double heterozygote *Apoer2::Vldlr* cerebella have a small number of ectopic Purkinje cells restricted to the zebrin II-immunonegative subset

**Figure 8:**
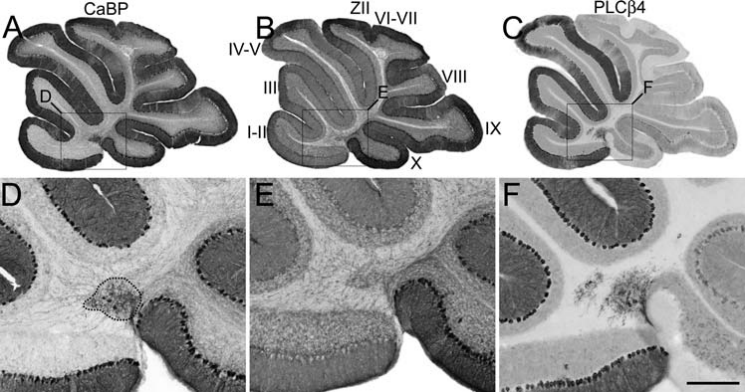
Cerebella from mice heterozygous for the *Apoer2* and *Vldlr* deletions have Purkinje ectopia restricted to a small subset of zebrin II-immunonegative Purkinje cells. Serial sagittal sections from adult cerebella immunoreacted with anti-calbindin (CaBP -A, E), anti-zebrin II (ZII-B, F), or anti-phospholipase C ß4 (PLCß4-C, G) antibodies reveal the presence of a small cluster of Purkinje cells (e.g. dotted circle–D) that fail to express zebrin II but do express PLCß4 (F). Roman numerals denote putative lobule assignments. Boxes in A–D indicate magnified areas. Scale bar in F = 1mm for A–C and 250 µm for D–F.

There is no evidence that single receptor heterozygotes (i.e., *Apoer2* ^+/−^ and *Vldlr* ^+/−^) have any Purkinje cell dispersal defects [25; data not shown]. However, cerebella from double heterozygotes (*Apoer2* ^+/−^:: *Vldlr* ^+/−^) reproducibly exhibited a subtle Purkinje cell ectopia (Fig. 8). The cerebella are normal in terms of size and lobulation and almost all Purkinje cells are positioned correctly in a tight monolayer throughout the rostrocaudal extent of the cerebellum (e.g., Fig. 8A). The single exception is a small pair of calbindin-immunoreactive ectopic Purkinje cell clusters located either side of the midline and midway between lobules I/II and X (Fig. 8A, D). Immunolabeling of neighboring sections with anti-zebrin II antibodies revealed these clusters to be zebrin II-immunonegative (Fig. 8B, E) and PLCß4-immunopositive (Fig. 8C, F).

## Discussion

In the cerebellum, external granular layer and cerebellar nuclear neurons secrete Reelin, which binds to Apoer2 and Vldlr receptors on Purkinje cells [21], [25], [44]. Binding activates intracellular tyrosine kinase cascades that require the phosphorylation of the intracellular adaptor protein Dab1 as well as several other small tyrosine kinases from the Src family among others [18], [28], [45], [46]. As a result Purkinje cells disperse from their clusters in the central cerebellum and migrate to the cerebellar cortex.

Mutations in the Reelin signaling pathway cause a phenotype in which most Purkinje cells fail to disperse to the cerebellar cortex. Mutant mice with this phenotype include *reeler* [22]–[24], [47], [48], Dab1 and its alleles (*disabled*–18, 19; *scrambler* and *yotari*-45), and mice lacking functional copies of both the *Apoer2* and *Vldlr* genes [25]. In addition to these reeler-like mutants, several “partial” mutants exhibit dispersal deficits restricted to specific Purkinje cell subsets. For example, *meander tail* and *rostral cerebellar malformation* mice each have ectopic Purkinje cells that are derived from the AZ population [48]–[51]. The *weaver* cerebellum also has a small population of ectopic Purkinje cells, in this case derived from discrete parasagittal stripes in the CZ [40]. Other mutant mice exhibit parasagittally-restricted ectopias. For instance, most zebrin II-immunonegative Purkinje cells are located in the central cerebellum of the *cerebellar deficient folia* (*cdf)* mouse whereas the zebrin II-immunopositive Purkinje cells disperse normally [41].

In the present report we show that a significant regulation of Purkinje neuron dispersal occurs at the level of the Reelin receptor.

Recent work examining neuron dispersal in the cerebral cortex similarly indicates that Apoer2 and Vldlr also play diverging roles in regulating neuronal migration during cortical development [51]. These findings are complementary to the current study because they reaffirm our observations of divergent effects on neuronal migration mediated by each individual receptor. Moreover this study also suggests that each reelin receptor is capable of mediating the dispersal of subsets of populations of neurons.

Our evidence demonstrates that Purkinje cell ectopia is restricted to parasagittal and/or transverse subsets of Purkinje cells in both *Apoer2* and *Vldlr* null cerebella. The analysis depends on the assumption that Purkinje cells reliably express their normal phenotypic antigenic markers when in ectopic locations. Considerable evidence supports this hypothesis. First, studies of cerebellar development have shown that Purkinje cells are already committed to their adult phenotype at around their time of birth in the 4^th^ ventricle (E10–E13: 6, 8) and that subsequent experimental manipulations cannot alter this [e.g., 53], [54], [31]: [reviewed in 11], [40]. Secondly, there are several examples of mutants in which Purkinje cells are ectopic but still express an appropriate phenotype (e.g., zebrin II-immunopositive/HSP25-immunopositive from the CZ in *weaver*–40; zebrin II-immunonegative/PLCß4-immunopositive Purkinje cells from the AZ in *cdf*–41). Thirdly, in complete dispersal mutants, ectopic Purkinje cells show a mediolateral striped patterning consistent with their normal adult phenotypes despite the fact that they fail to disperse to the cerebellar cortex (*reeler*–55; *disabled*–19; *scrambler*–43, 45; 57). It has been previously reported that Reelin signaling may affect glial morphology [56]. It will be interesting to explore if Bergman glia were affected in the either *Apoer2* or *Vldlr* null cerebella.

The ectopia in *Reelin* receptor mutants takes two forms-in some cases, the failure to disperse is complete and the Purkinje cells form reproducible, tightly packed clusters in the cerebellar core (e.g., *Apoer2* null-Fig. 2J, H, L; *Vldlr* null–Fig. 5D, E; *Apoer2::Vldlr* double heterozygote–Fig. 8D–F). In other cases, the embryonic cluster disperses but migration is defective and the Purkinje cells end-up trapped in the white matter tracts or granular layer (e.g., *Apoer2* null-Fig. 3M–O; *Vldlr* null–Fig. 5G–I). Although not mutually exclusive, there are two ways to account for the different phenotypes-reflecting either the distribution of the receptors or differential sensitivity to receptor loss.

Support for a model where Apoer2 and Vldlr are selectively expressed in subsets of Purkinje cells comes in part from recent reports indicating that these receptors are differentially expressed in various neural populations including cortical neurons and Purkinje cells [58], [59]. Evidence for parasagittally-restricted expression of both receptors in Purkinje cells is presented in the Allen Brain Atlas (www.brain-map.org). While the expression pattern for each receptor in the adult cerebellum is consistent with the ectopia that we observed in the mutants, it will be interesting to explore if this restricted expression pattern is present in Purkinje cells during development while dispersal is occurring. Interestingly, humans homozygous for a *Vldlr* deletion show profound Purkinje cell migration defects [60]. In this model, one subset of Purkinje cells would only express the Apoer2 receptor. These cells would completely fail to disperse and remain in compact embryonic clusters in the central cerebellum of the *Apoer2* null (e.g., the zebrin II-immunonegative/PLCß4-immunopositive cluster: Fig. 2J, H, L; Fig. 3P, Q, R). However, in the *Vldlr* null cerebellum, these Purkinje cells would disperse normally. A second group of Purkinje cells would express Vldlr but not Apoer2. In the *Vldlr* null cerebellum, these cells would remain in embryonic clusters (e.g., the zebrin II-immunonegative/PLCß4-immunopositive cluster: Fig. 4D, E; Fig. 5I–L), whereas they would disperse normally in the *Apoer2* null. The third class of Purkinje cells would require both Apoer2 and Vldlr to ensure their proper dispersal, would be sensitive to the deletion of either receptor and therefore disperse poorly in either null mutant. Poor dispersal would present as stalling *en route* in the intralobular white matter or granular layer of the cortex (e.g., the intralobular ectopic Purkinje cells in the *Apoer2* null–e.g. Fig. 3M–O; in the *Vldlr* null-5G–I).

No simple correlation between adult Purkinje cell antigenic phenotype and the ectopia observed in the three mutants is apparent. The *Apoer2* null is the most straightforward: the tight Purkinje cell ectopic clusters are all zebrin II-immunonegative/PLCß4-immunopositive (e.g. Fig. 3P–R). The same is the case for the double *Apoer2:: Vldlr* heterozygote (Fig. 7D–F). However, in the *Vldlr* null both zebrin II-immunopositive (e.g., Fig. 5H) and zebrin II-immunonegative (Fig. 5F) Purkinje cells fail to disperse. The phenotypes of the intralobular (poorly-dispersing) Purkinje cells on the other hand are a mixture of zebrin II-immunopositive (e.g. *Apoer2* null–3N; *Vldlr* null–5H) and zebrin II–immunonegative (e.g. *Apoer2* null–3O; *Vldlr* null–5I) Purkinje cells. Moreover, there should be no overlap between the Purkinje cells that fail to disperse from clusters in the two nulls, but this is not always the case. For example, zebrin II-immunonegative/PLCß4-immunopositive ectopic Purkinje cells form a tight cluster in the anterior cerebellum of the *Vldlr* null cerebellum (Fig. 4D, E, F). Ectopic clusters are also seen in the same location and with the same phenotype in the *Apoer2* null (Fig. 2H, J, L) and double *Apoer2::Vldlr* heterozygote cerebella (Fig. 8D–F: however the clusters are progressively smaller for each mutant -*Vldlr*>*Apoer2*>*Apoer2::Vldlr* double heterozygote). The simplest explanation for this observation is that zebrin II-immunonegative cells comprise two or three subgroups, each of which expresses a different receptor combination (Apoer2, Vldlr, or both). However, if indeed the Purkinje cells ectopic in *Apoer2* nulls are a subset of those ectopic in *Vldlr* then this observation is not easy explained simply by receptor distributions. One possibility is that co-ectopia arises non cell-autonomously. For example, wild type <-> *scrambler* chimeras reveal a community effect wherein Purkinje cells with defective Reelin signaling negatively influence the dispersal of wild type cells [61].

It will be interesting to determine the interplay between Apoer2 and Vldlr receptors and its role in neuronal migration. It is unclear if each receptor regulates the dispersal of unique populations of cells, if they have a synergistic relationship, or some combination of the two, especially as Hack and colleagues [51] have shown that these receptors regulate the dispersal of unique populations of neurons in the cortex. Our data similarly suggests that each receptor is capable of regulating the dispersal of both unique and overlapping unique Purkinje cell subsets.

Selective ectopia can also be explained by postulating that the distribution of Apoer2 and Vldlr in Purkinje cells is homogeneous and selective ectopia is due to the differential sensitivity to the mutation of Purkinje cell subsets. There are many examples in which an entire population of Purkinje cells expresses a mutant protein but only a subpopulation is adversely affected. For example, in mouse models of Niemann-Pick type C disease, all Purkinje cells express the mutant NPC1 protein but zebrin II-immunonegative Purkinje cells are far more susceptible to its effects [15]. Similarly, all Purkinje cells in the *tottering* mouse express a mutant alpha 1a calcium channel but only the zebrin II-immunonegative population dies [62]. Patterned Purkinje cell death also occurs in *lurcher* (Lc/+), an ataxic mouse strain with gross cerebellar deficits due to a gain-of-function point mutation in the orphan delta 2 glutamate receptor gene (GluRδ2/Grid2: 63–65). Possible roles for Apoer2 in apoptotic and excitotoxic neuronal death are discussed in [69]. There are precedents for these selective effects in dispersal mutants as well. For example, in the *weaver* mouse, in which all Purkinje cells express a mutated version of the inwardly rectifying K+ channel *girk2* [67], Purkinje cell ectopia is restricted to a small subset of HSP25-immunopositive Purkinje cells from the CZ [40]. Similarly, the *Catna2* gene, encoding for alpha-N-catenin, is truncated in the *cdf* null mutant [68], [69] but although *Catna2* is expressed in all Purkinje cells only those that are zebrin II-immunonegative are ectopic [41]. From this perspective, Purkinje cells in different stripes and transverse zones would each respond to each receptor mutation differently-unaffected, partially affected (i.e., disperse poorly) or unable to disperse. Further research will be required to parse out the exact roles of each receptor in regulating neuron dispersal, both in the cerebral cortex and the cerebellum.

Finally, we have observed a significant reduction compared to wild type in the length of several individual cerebellar lobules as well as in the total lengths of both *Apoer2* and *Vldlr* null cerebella (Fig. 1). Such reductions can be explained by changes in cell numbers as a result of apoptosis or reductions in neurogenesis. However, it is unclear if either of these processes contributes to the change in cerebellar size or if there is even a change in Purkinje cell numbers in the mutants.
